# Advances in Biomass-Derived and Biodegradable Polymer Materials: Synthesis and Application

**DOI:** 10.3390/ma19050879

**Published:** 2026-02-26

**Authors:** Jongbok Lee

**Affiliations:** Department of Biological and Chemical Engineering, Hongik University, Sejong 30016, Republic of Korea; jlee0917@hongik.ac.kr

## 1. Introduction

Conventional plastics have profoundly influenced modern society by enabling durable consumer goods, protective packaging, medical devices, and countless industrial applications [1]. Their widespread use, however, has resulted in unintended consequences. Most petroleum-based plastics are neither biodegradable nor efficiently recycled, which leads to accumulation in landfills, fragmentation into microplastics, contamination of oceans and soils, and greenhouse gas emissions throughout their life cycles [2]. As these issues continue to grow, the scientific community has increasingly turned to biodegradable and biomass-derived polymers as essential tools in the movement toward sustainability.

Although often considered together, biodegradable and bio-based polymers represent distinct but complementary concepts [3]. Biomass-derived polymers are synthesized from renewable carbon sources such as agricultural residues, forestry byproducts, plant-derived sugars, or marine biomass, thereby reducing dependence on fossil resources. Biodegradable polymers, in contrast, are designed to undergo chemical and enzymatic or microbial breakdown into low-molecular-weight species that are ultimately converted to carbon dioxide, water, methane, and biomass under defined environmental conditions [4]. The greatest promise would arise when both attributes combine, enabling materials that originate from renewable feedstocks and are subsequently reintegrated into natural cycles after use.

Importantly, the motivation for developing biodegradable and biomass-derived polymers extends beyond mitigating plastic pollution or achieving carbon neutrality. These materials enable emerging technological approaches that are difficult or impossible to realize using conventional, non-degradable plastics. In biomedical engineering, for example, biodegradable polymers are essential to the development of temporary, self-degrading in-body materials and systems, including resorbable sutures, orthopedic fixation systems, drug-delivery systems, and transient bioelectronics [5,6,7,8]. Such systems are designed to perform a precise function over a defined period and then safely degrade, eliminating the need for secondary surgical removal and minimizing long-term complications.

Similarly, biodegradable polymers are increasingly explored in implantable sensors, transient diagnostic systems, and controlled-release therapeutic systems, where degradation kinetics are engineered to match healing processes or pharmacological timelines [8,9]. In agriculture and environmental engineering, controlled degradation materials enable seed coatings, soil conditioning films, and nutrient release systems that disappear after fulfilling their role [10]. Even in electronics and soft robotics, bio-derived polymers are being investigated as substrates for transient or environmentally dissolvable devices, addressing the growing problem of electronic waste [11].

These advanced applications highlight a critical shift in perspective. Biodegradability is no longer merely viewed as an environmental compromise or a disposal strategy, but as a functional design feature that enables precision, safety, and adaptability. As a result, modern biodegradable polymer research must simultaneously address molecular design, functional performance, degradation control, and system-level integration.

The papers collected in this Special Issue reflect this expanded vision. They range from the molecular engineering of bio-based composites and reinforced natural materials to functional coatings, intelligent packaging systems, biomedical interfaces, and biomass valorization strategies. To clarify their collective contributions, the works are discussed within two thematic perspectives that focus on advances in sustainable polymer design and composite engineering, as well as the practical application of bio-derived polymers. They demonstrate how biodegradable and biomass-derived polymers are playing an important role in advancing both environmental sustainability and next-generation technologies.

## 2. Sustainable Polymer Design and Composite Engineering

Several contributions in this Special Issue address the fundamental challenge of reconciling biodegradability with mechanical performance and processing stability. A representative example is the study on poly(3-hydroxybutyrate)–polyurethane biocomposites enriched with urea [12]. Poly(3-hydroxybutyrate), while fully biodegradable and biosynthesized, is well known for its brittleness and narrow processing window, which limit broader application. By incorporating flexible polyurethane segments, the authors significantly improved ductility, while the introduction of small amounts of urea modified intermolecular interactions through hydrogen bonding. Mechanical testing revealed that the addition of approximately 1 wt.% urea increased stiffness relative to polyurethane-modified P3HB, partially compensating for softening effects, while composting experiments demonstrated accelerated mass loss, reaching ~40% under controlled conditions. Importantly, excessive urea content deteriorated microstructural homogeneity and mechanical integrity, demonstrating the importance of precise formulation control. The work exemplifies how biodegradable materials can be designed to balance in-use performance with functional end-of-life behavior, particularly for short-life agricultural products such as seedling containers.

The reinforcement of renewable materials was further explored through the in situ polymerization within wood, with Scots pine recovered from demolition timber serving as a key example [13]. In this study, monomer solutions were introduced into the wood and polymerized under controlled conditions, and the resulting materials were evaluated primarily through mechanical testing. The authors reported substantial increases in compressive strength and stiffness after treatment, with the most pronounced relative improvements observed in the least dense material, particularly reclaimed pine. These changes were accompanied by significant increases in density and weight percent gain, indicating extensive polymer uptake within the wood structure. While no direct chemical or microscopic characterization was performed, the mechanical response suggests that polymer incorporation within the porous architecture of the wood plays a central role in the observed reinforcement. Rather than altering the native structure of the material, the approach makes use of the existing anatomical characteristics of wood to enhance performance, suggesting a practical strategy for upgrading low-quality or reclaimed timber without relying on conventional fiber–matrix composite concepts.

Processing efficiency and sustainability were examined in a study on wood–plastic composites produced from Pinus sylvestris following hot-water extraction pretreatment [14]. The authors analyzed compaction behavior and energy consumption, showing that extraction altered compressibility and reduced the energy required during densification without compromising mechanical performance. These findings are particularly relevant for large-scale manufacturing, where energy demand often dominates environmental impact. By addressing both material formulation and processing efficiency, the study emphasizes the importance of evaluating sustainability across the full production chain.

A broader conceptual perspective on biodegradable polymer design is provided by a comprehensive review of biodegradable plastics, with particular emphasis on aliphatic polyesters such as PLA, PHAs, PBS, PBAT, and PCL [15]. The review discusses how crystallinity, molecular weight, copolymer architecture, and blending strategies govern both mechanical properties and degradation behavior. While significant progress has been made in tailoring performance, the authors emphasize unresolved challenges related to recycling incompatibility, infrastructure limitations, and inconsistent degradation behavior outside industrial composting environments. This analysis places individual experimental advances within a broader system-level context, highlighting that material design must align with realistic end-of-life pathways to achieve sustainability.

Overall, the papers in this section demonstrate that biodegradable and bio-based polymer development has progressed beyond simple material substitution. Instead, it increasingly focuses on structure–property–lifetime relationships, where degradation behavior is engineered alongside mechanical and processing performance.

## 3. Functional Applications of Biodegradable and Bio-Based Polymers

The second group of papers illustrates how biodegradable and bio-based polymers are being employed in application-driven contexts where functionality and controlled lifetime are equally important. In food preservation, chitosan-based coatings prepared using kombucha as a natural acidic medium were applied to fresh produce [16]. Kombucha, containing organic acids generated through fermentation, served as an alternative to conventional acids for dissolving chitosan, enabling film formation without synthetic additives. When applied to red peppers, the coatings reduced microbial growth, slowed moisture loss, and helped maintain firmness during storage. These effects were attributed to the combined barrier properties and inherent antimicrobial activity of chitosan, demonstrating that bio-derived coatings can actively extend shelf life while remaining edible and biodegradable.

Smart packaging concepts were explored through electrospun nanofibers incorporating anthocyanin extracts dissolved in natural deep eutectic solvents (NADES) [17]. By addressing formulation and processing limitations, the authors produced continuous nanofibers capable of responding visibly to pH changes. The fibers exhibited distinct color transitions when exposed to volatile compounds generated during food spoilage, confirming their potential use as freshness indicators. This work demonstrates that biodegradable polymers can support sensing and signaling functions, extending their role beyond passive containment.

Biomedical applications were examined in a comprehensive review of chitosan-nanometal coatings designed for antimicrobial surfaces [18]. The review covered fabrication methods such as dip coating, electrophoretic deposition (EPD), and layer-by-layer (LBL) assembly, as well as the incorporation of metallic nanoparticles, including silver, zinc oxide, copper, and gold. These hybrid coatings consistently exhibited enhanced antibacterial performance against both Gram-positive and Gram-negative bacteria, while maintaining favorable interactions with mammalian cells in many reported cases. At the same time, the authors highlighted unresolved challenges related to controlling ion release, avoiding cytotoxicity, and meeting regulatory requirements, particularly for implantable applications. The review emphasizes the need to balance antimicrobial efficacy with long-term biological safety when biodegradable polymers are combined with active inorganic components.

Environmental and resource-oriented applications were represented by a detailed physicochemical characterization of forestry byproducts derived from pine and acacia species [19]. The study examined differences in cellulose, hemicellulose, lignin, extractives, ash content, and thermal stability across various biomass fractions, including branches, leaves, and needles. These compositional differences were shown to influence adsorption behavior and thermal degradation profiles, with branch-derived materials exhibiting higher structural carbohydrate and lignin content and improved performance for certain functional uses. By linking biomass composition to material behavior, the work provides a scientific basis for selectively valorizing forestry residues as functional materials or precursors, supporting the view that biomass streams can be engineered resources rather than disposal burdens.

Across these applications, biodegradability is considered not simply an environmental attribute but a design feature that enables controlled functionality, biological integration, and system-level sustainability.

## 4. Conclusions

The contributions presented in this Special Issue demonstrate that biodegradable and biomass-derived polymers have evolved into strategically engineered material systems capable of addressing both environmental challenges and emerging technological demands. Rather than serving solely as replacements for conventional plastics, these materials increasingly function as key contributors to new design concepts, where controlled lifetime, biological compatibility, and environmentally benign degradation are intrinsic advantages.

In this Special Issue, sustainability is treated not as a limitation but as a driver of innovation. By tailoring polymer architecture, composite interfaces, and degradation behavior, researchers are creating materials that maintain mechanical integrity and functional performance during use, yet undergo predictable and safe transformation afterward. This dual requirement is particularly evident in applications such as antimicrobial coatings, smart food packaging, biodegradable agricultural products, and bio-integrated medical systems.

It is increasingly recognized that biomass-derived feedstocks exhibit considerable structural richness and chemical diversity, rather than serving as homogeneous raw materials. Detailed characterization of forestry residues and natural polymers enables rational selection and design, facilitating the development of materials that align performance requirements with ecological responsibility. Such approaches support circular-economy models in which waste streams are converted into functional resources.

Looking forward, the importance of biodegradable and bio-based polymers will continue to expand as society demands materials that are not only sustainable but also intelligent, adaptive, and compatible with biological systems. Future advances will depend on integrating molecular-level design with system-level considerations, including degradation environments, regulatory frameworks, waste management infrastructure, and life-cycle assessment. Interdisciplinary collaboration across chemistry, materials science, biology, medicine, environmental engineering, and policy will be essential.

Ultimately, biodegradable and biomass-derived polymers are expected to define a new class of materials whose value lies not only in what they replace but in what they make possible. By enabling technologies such as self-degrading medical devices, transient functional systems, and environmentally responsive materials, these polymers represent a foundational component of a more sustainable, efficient, and technologically advanced society. The studies in this Special Issue provide compelling evidence of this transition and offer a forward-looking perspective on the future of sustainable polymer science.
